# Gravity and Electrostatic Separation for Recovering Metals from Obsolete Printed Circuit Board

**DOI:** 10.3390/ma15051874

**Published:** 2022-03-02

**Authors:** Camila Mori de Oliveira, Rossana Bellopede, Alice Tori, Giovanna Zanetti, Paola Marini

**Affiliations:** 1 Department of Environment, Land and Infrastructure Engineering (DIATI), Politecnico di Torino, 10129 Turin, Italy; camila.morideoliveira@studenti.polito.it (C.M.d.O.); giovanna.zanetti@polito.it (G.Z.); paola.marini@polito.it (P.M.); 2 OSAI Automation System S.p.A., 10010 Parella, Italy; a.tori@osai-as.it

**Keywords:** PCB, recycling, metal recovery, mechanical pre-treatment

## Abstract

This study proposed an evaluation of enrichment processes of obsolete Printed Circuit Boards (PCBs), by means of gravity and electrostatic separation, aiming at the recovery of metals. PCBs are the most important component in electronic devices, having high concentrations of metals and offering a secondary source of raw materials. Its recycling promotes the reduction in the environmental impacts associated with its production, use, and disposal. The recovery method studied started with the dismantling of the PCB, followed by a comminution and granulometric classification. Subsequent magnetic, gravity, and electrostatic separations were performed. After the separations, a macroscopic visual evaluation and chemical analysis were carried out, determining the metal content in the concentrate products. The results obtained from gravity separation showed a product with metallic concentrations of 89% and 76% for particle sizes of 0.3–0.6 mm and 0.6–1.18 mm, respectively. In electrostatic separation, the product obtained was 88% for the lower particle size (<0.3 mm) and 62% for particles sizes >1.18 mm.

## 1. Introduction

It is difficult to imagine life today without technology, especially in the pandemic scenario, in which online meetings, classes, and appointments have become routine. Therefore, technology has taken up space, incorporating indispensably into everyday life very quickly and intensely.

The current digital context generates an extensive number of electronic products and, due to the advancement of technology, Electrical and Electronic Equipment (EEE) becomes obsolete faster and, consequently, their disposal also increases. Viewing them from the perspective of an exploitation potential for use can promote urban mining [1].

Urban mining, in contrast to traditional mining, consists of the process of obtaining raw materials derived from waste, being recycled and reused by the industry. The materials obtained from the recycling of the devices are called secondary raw materials [2].

The electronics industry is estimated to generate 57.4 million tons of waste electrical and electronic equipment (WEEE) in 2021, which represents 7.6 kg of WEEE per inhabitant, and in 2019, only 17.4% of the generated amount was officially documented as properly collected and recycled [3]. According to that same source, by the end of 2030, the mark of 74.7 million tons of WEEE is estimated to have been reached worldwide. 

An effective recycling of these materials is essential to keep them available for the manufacturing of new products, conserving natural resources, and being a great contribution to the circular economy, removing waste from its disposal and reinserting it in the production cycle. Thus, the proper management of electronic waste is essential to guarantee access for future generations of electronic products, to preserve natural resources and human health, to protect working conditions, to reduce the environmental impacts associated with production, and to use and dispose of electronic equipment [4].

Plenty of research has been carried out in recent years to characterize the electronic waste generated, consisting mainly of household appliances, computers, televisions, and other goods that are damaged or broken [5]. WEEE encompasses up to 69 elements from the periodic table, becoming a diverse and complex type of waste having both hazardous and nonhazardous compounds [3]. The presence of metals, such as copper, gold, silver, and critical raw materials, such tantalum, makes the WEEE extremely attractive to recovery. Some elements have concentrations significantly higher than those usually found in corresponding mineral ores [6]. 

Printed Circuit Boards, also known as PCBs, are generally not visible, but they are part of everything that involves technology. They represent about 3% by weight of WEEE and, taking into account that their composition can reach approximately 40% of metals, the recycling of obsolete PCBs has a high economic importance [2]. A common structure of a PCB is made of layers of glass fibers and copper clads, usually held together by halogenated epoxy resins (HERS) or brominated epoxy resin (BES) in which electrical components (e.g., resistors, capacitors, and inductors) are soldered onto the top layer [7,8]. They can be structurally classified according to the number of layers: single-sided, having a conducting layer (copper) on one side; double-sided, having a conducting layer on both sides; and multi-layers, having metallized holes to connect different layers. The heterogeneous PCB composition hinders the process of recovering the materials, making it slow and expensive [8]. Thus, much research has been developed to optimize the efficiency of a sustainable recycling process of these components.

In recent years, the mainstream of the recycling approaches of PCBs has focused on chemical process approaches [9], including co-pyrolysis [10], hydrometallurgy with nitric acid [11], and bio-metallurgical processes by biosorption and bioleaching [12]. These processes are very time-consuming, high energy-demanding, and may release significant pollutants into the atmosphere [13]. Despite the progress of these chemical techniques, at present, there is a lack of studies focusing on sophisticated solutions to obtain physicomechanical improvements. Therefore, there is still a demand to identify low-cost and eco-friendly methods to improve the reach of a high concentration of metals and decrease the rate of metal loss during these operations.

In this context, the objective of the article is to evaluate green mechanical pre-treatments available for the sustainable recycling of PCBs, intending to obtain highly concentrated material before the chemical recovery processes. Treatment through eco-friendly processes will contribute not only to the solution from an environmental point of view, but also from an economical one, to increase the metal recovery rate during the operations and establishing an advanced industrial recycling sector.

To achieve that, the characterization of an obsolete PCB and the evaluation of the efficiency of gravimetric and electrostatic separation was performed. A pre-treatment composed of comminution, granulometric, and magnetic separations was performed before. Then, gravity separation by means of a shaking table and electrostatic separation by means of corona electrostatic separation were accomplished. To ascertain the metal content existing in the concentrated fractions after applying the treatments, samples were collected for visual analysis with a macroscope, and chemical analysis by inductively coupled plasma–optical emission spectroscopy was performed.

## 2. Materials and Methods

### 2.1. Materials

Printed circuit boards are composed of ceramic, polymers, and metals. The composition can vary significantly depending on the model of equipment and the age of the boards. For example, before the year 2003, equipment was fabricated containing solders with tin and lead alloys; however, after the Directive 2002/95/CE, where there is a restriction on the use of these substances, there is currently is a trend toward replacing this with SnAgCu solder alloys, which are considered “lead-free” [1].

PCBs typically consist of more than 60 different types of elements, having a content of base metals (Cu, Zn), precious metals (Au, Ag, Pd), and heavy metals even higher than those in natural minerals [8]. The chemical composition is also used to distinguish the PCBs among them, with scrap being a low-grade PCB that has a low concentration of precious metal (i.e., gold) [14]. 

Due to this variation in the composition, the material characterization step is essential to define the treatments aiming to recover and recycle materials. Several studies have been performed on printed circuit boards, and part of these studies also involved the characterization of the boards. As many recyclers receive a mix of low- and high-grade PCBs, not having a specific composition selection [9], a data compilation from several authors [15,16,17,18,19,20,21,22,23,24,25,26,27,28,29,30] and different types of PCBs (computers, smartphones, etc.) have been elaborated (Table 1) in order to obtain a mean value (and a standard deviation) for each metal of the PCBs, thus reaching an average reference value of PCB composition. The results differ, as the boards come from different products and periods. On average, 34.7% of a PCB’s mass is metals. Cu is the metal with the highest concentration present in printed circuit boards, an average of 21.44%. Other metals with a significant mean percentage weight are Al (3.02%), Fe (3.28%), and Sn (3.14%). The quantities of valuable metals are significant considering, for example, that the average rate of gold in PCBs (0.04%) is higher than that in raw gold ore (0.0005%) [19]. It is possible to notice that some elements have a mean and standard deviation about the same size, such as tantalum, and the paucity of data gives a very skewed distribution, having a grossly inflated standard deviation. 

It is also possible to observe the commodity metals market prices, from the London Metal Exchange [31], for 2 November 2021. Combining the average composition with the price, it is possible to quantify the economic value of the obsolete PCBs. These data reveal that a ton of waste PCBs can reach up to USD 60,000. Copper, representing 21.4% by weight, creates around of 3.5% of the product price. Another highlight is for the precious metals, gold or palladium, whose mass in a PCB represents less than 1%; however, from an economical point of view, they can represent more than 90% of the price market.

The printed circuit board used in this research was supplied by OSAI Automation System S.p.A, Parella, Italy, and represented a PCB of a server. It has a multi-layer structure, and its total weight, containing all the electronical components, was about 1.8 kg. Each electronical component was recognized and analyzed on the SEM (FEI Company, Hillsboro, OR, USA), coupled with an energy dispersive X-ray spectroscopy EDS detector (EDAX (Ametek Inc.), Mahwah, NJ, USA), to identify the chemical elements composition. The analysis showed that a significant number of metal elements of the periodic table were present: Sn, Pb, Au, Cu, Ni, Pd, Ag, Al, Fe, Ta, Ba, and Mn. The lack of data for some metals (e.g., barium and manganese) in Table 1 may be explained by the very low concentrations. 

### 2.2. Methods

**Figure 1:**
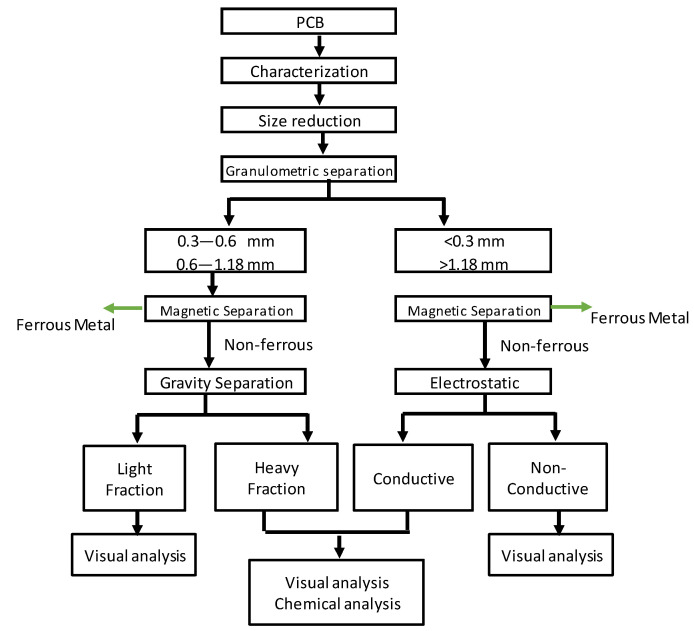
Flowsheet of the process applied in this study.

In order to recover metals from the obsolete PCB, the methodology presented in Figure 1 was applied. Prior to the mechanical separation, a preliminary work was carried out consisting of the identification of the PCB and its constituent composition. Then, the dismantling of the plates followed by a comminution and granulometric classification were carried out. Next, according to the particle size obtained by sieving, magnetic and gravity methods were used for the medium classes, while magnetic and electrostatic methods were used for the coarser and the finest classes. A visual evaluation of the quality of the products by means of a macroscope and chemical analyses were carried out, in order to assess the potential of the method in the recovery of metals present in printed circuit boards.

- Disassembly and size reduction

The disassembly of the board was carried out manually in the Raw Materials Laboratory of Politecnico di Torino, Turin, Italy using different types of tools, in which elements of greater volume and without interest such as liquid electrolytic capacitors and the central process unit were removed. About 80 g of material was removed.

Subsequently, the first board was cut into pieces with a maximum size of 2.5 × 4.0 cm, enabling an adequate feed to the shredding operation performed by means of a cutting mill RETSCH SM100 (Retsch GmbH, Haan, Germany) with a rotor made up of three nonaligned blades, whose action fragments the material introduced. The size reduction is an important phase of mechanical processing because it allows the release of materials from printed circuit boards, enabling the optimization of metal recovery.

- Granulometric classification

Considering the heterogeneous composition of the PCB, in order to minimize errors, the fragments generated should be classified granulometrically, facilitating the characterization of materials through chemical analysis, and enabling the identification of fractions of concentrated metals and nonmetals. The fragmented material was classified by means of a sieve shaker model FTL-0200 (OMM, Busnago, Italy), and a series of standard ASTM sieves (Controls, Milano, Italy) (1.18 mm; 0.6 mm, 0.3 mm and bottom) were used. The samples were weighed in order to calculate the retained mass in each granulometric range.

After these steps, which aimed to prepare the material for separation tests, the enrichment operations began.

- Magnetic separation

In magnetic separation, the materials can be classified according to their responses to the magnetic field. There are ferromagnetic materials, which are strongly attracted by the magnetic field; paramagnetic materials, which are weakly attracted; and diamagnetic materials, which are repulsed [33]. In this study, this separation was performed manually, using a AlNiCo magnet with an intensity of 1 T. The experimental parameters included material particle size and distance between magnet and fragments, established at 1 cm. The products obtained were a ferromagnetic and a nonferromagnetic portion.

- Gravity separation

Separation by gravity allows the classification of materials based on their different densities. In these processes, particles are separated thanks to different sedimentation velocities when falling into a fluid (air, water). This speed will simultaneously depend on its density and size. In the present work, the separation was performed by a wet shaking table model Krupp (Humboldt Wedag GmbH, Köln, Germany) on particle classes 0.6–1.18 mm and 0.3–0.6 mm. The adjustable parameters of the device included its inclination, the frequency of movement, the water flow, and the feed speed. The frequency was 300 cycles per minute, with 1° of inclination angle and a water flow of 10 L/min. In the concentrate collection and tailing discharge areas, gutters were placed, with a partition separating these same areas, to collect the products resulting from the separation. After the passage, the products collected in the different fractions were filtered and placed to dry in an oven, at 40 °C. The products obtained were classified as heavy fraction (metals concentrate) and light fraction (tailing).

- Electrostatic separation

The corona electrostatic separation was carried out on particle size classes >1.18 mm and <0.3 mm using the separator Dings Coronatron (Prodecologia, Rivne, Ukraine). This process is based on the electrical conductivity of some elements. In this way, a fraction rich in conductive metals, such as copper, and another one consisting of polymeric and ceramic materials were obtained [1]. After some tests, it was established to perform two passages. The first passage with a voltage of 20 kV and a rotation speed of 30 Hz was performed. After that, a second passage, with the same parameters, was performed only to the conductive product obtained from the first passage, increasing the quality of the products.

- Visual characterization

After the mechanical treatments, an inspection of the quality of all products was performed by means of a visual analysis using the optical macroscope Leica/Wild M420 (Leica Microsystems, Wetzlar, Germany).

- Chemical characterization

On the metal-concentrated products, a chemical analysis using the Inductively Coupled Plasma–Optical Emission Spectrometry instrument (Perkin Elmer, Optima 2000DV, Waltham, MA, USA) was carried out.

The chemical analysis was divided into microwave digestion and optical spectroscopy. For each product, two samples were performed.

The metals leaching was obtained by the microwave digestion system Milestone MLS-1200 Mega (Milestone, Sorisole, Italy) laboratory unit with aqua regia (nitric acid 65%/hydrochloric acid 37%) and HF. The analyzed product was added in the proportion of 0.25 g for 6 mL of aqua regia and 1 mL of HF. The mixture was then subjected to microwave heating to complete the digestion. After that, the content of each tube was filtered directly into a 50 mL volumetric flask, which was brought to volume with distilled water.

Each volumetric flask was analyzed by ICP–OES (Perkin Elmer, Optima 2000DV, Waltham, MA, USA). A calibration line was prepared at increasing concentrations, containing the following metals: lead, copper, tantalum, gold, tin, nickel, and aluminum, which was used to determine the concentration of metals in each sample.

## 3. Results and Discussion

During the treatment steps, losses in material are common. In the following sections, the yield of each product is reported in terms of percentage disregarding the losses (which are 15% of the PCB total weight).

### 3.1. Size Reduction and Classification

The size reduction performed by the cutting mill was performed in two stages. The screen used was 2 mm. To enhance the liberation of particles, the >1.18 mm size fraction size was re-shredded. A significant release of fine particles was observed. When compared to the initial feed, a loss of 11% was already accounted. 

**Figure 2:**
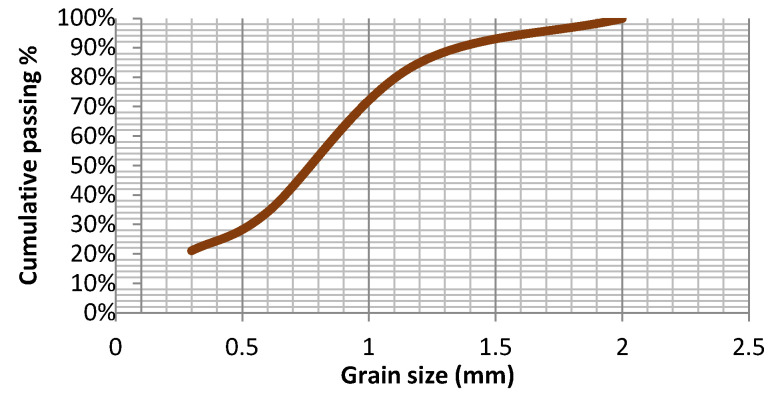
Particle size distributions of the PCB after size reduction.

The grain size classification step provided the separation of the previously fragmented material in different classes. It can be observed from the Figure 2 that only about 16% of the material mass was obtained in particle sizes over 1.18 mm. An amount of 50% of material was particles between 0.6 and 1.18 mm, 13.2% of material was particles between 0.3 and 0.6 mm, and 21% of material was particles less than 0.3 mm. 

### 3.2. Magnetic Separation

The magnetic separation was the first separation step to be performed as it is a common and easy classification that isolates the magnetic particles. Table 2 shows the results for each class size fraction. 

The greater amount of ferromagnetic material was observed in the >1.8 mm fraction, which may be due to the element being used in larger components, such as supports, and remains in the larger fractions due to its mechanical properties, making it more difficult to grind than polymeric materials. 

Table 2 shows that around 2.3% of the total product weight obtained from the separations was separated in this step. The presence of copper in the magnetic products was also observed, as despite not being magnetic, it is the predominant element in PCBs and its presence in these fractions can be justified due to them being dragged by iron and nickel particles when attracted by the magnet. 

Even though a low concentration was found, the main objective of this treatment was to separate iron, having a higher efficiency in the obtaining of copper in conductive fractions from electrostatic separation.

### 3.3. Gravity Separation

The fraction 0.6–1.18 and 0.3–0.6 mm of nonferrous material went to the wet shaking table separation. 

Regarding the grain size particles >1.18 mm, despite a possible efficient separation, it was not carried out, because the volume of material was not enough to perform this treatment. Nonetheless, the particles <0.3 mm were not performed, due to the difficult collection of products after separation. Both were taken to electrostatic separation.

The products obtained were weighed and the data are shown in Table 3. The yield of each product in terms of percentage was also calculated. Assuming that the heavy-fraction products obtained were mostly made up of metallic elements, the efficiency of the treatment was obtained through the weight in percentage of the products. The 0.6–1.18 mm class was the one with the highest efficiency of 59%, followed by the granulometric class 0.3–0.6 mm with 33%. 

### 3.4. Electrostatic Separation

From Table 4, as expected, it is possible to observe a small amount of conductive product for the finest grain size (<0.3 mm). This low efficiency can be explained due the fact that metals have a high mechanical strength and the fine material resulting from comminution mainly consisted of fiberglass. 

For the class size >1.18 mm, the conductive product obtained was copper grains attached to the fiberglass and the epoxy resin. The content of conductive elements (copper) reached more than 64% (in mass). The nonconductive product had an appearance with a predominance of polymer and fiberglass. 

### 3.5. Visual Characterization

**Figure 3:**
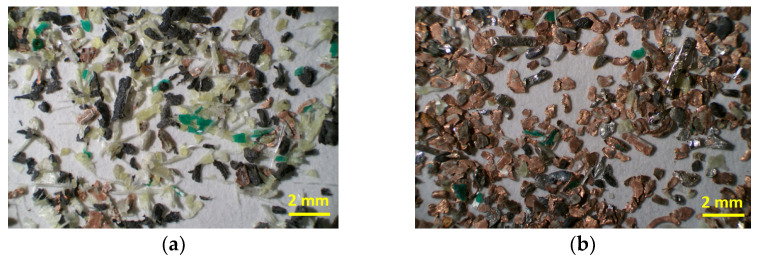
Light fraction (**a**) and the heavy fraction (**b**) resulting from gravity separation for the class size of 0.3–0.6 mm.

For the gravity separation, the macroscope visual observation of the products allowed us to conclude that the heavy fraction (Figure 3b) was rich in metallic elements (many filaments from electronic connectors) and had a low quantity of nonmetallic elements. However, metallic elements were also visible in the light fraction (Figure 3a), but in reduced amounts. In both cases, the separations were satisfactory.

**Figure 4:**
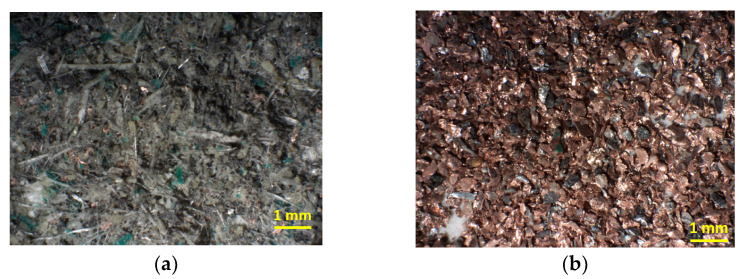
Nonconductive (**a**) and conductive (**b**) products resulting from electrostatic separation for the class size <0.3 mm.

Nonetheless, for the electrostatic separation, it can be seen in Figure 4b that the product obtained was composed mostly of metals in copper color and some in gray/silver color. On the other hand, the nonconductive fraction, as can be observed in Figure 4a, was mainly made up of a mix of resin and fiberglass, having a green dark color.

### 3.6. Chemical Characterization

Table 5 shows the mean concentrations of metals in the metal-enriched products resulting from gravity (heavy fraction) and electrostatic separation (conductive), and it is possible to also observe the yield of each product in terms of percentage considering all PCB weight. The chemical analysis was performed to determine the concentrations of Cu, Pb, Ta, Au, Sn, and Al. Ni was found in connectors having a double layer metallic coating with gold; therefore, to evaluate the liberation between Ni and Au, this element was also included in the chemical analysis whose results showed a presence of nickel in all the grain size fractions. 

From Table 4, it is evident that the finer the particle size resulting from the comminution, the higher the probability of obtaining a greater amount of “free grain,” and therefore a higher efficiency in the separation processes. 

Copper is the metal with the most abundant concentrations in the PCB, having been found with a content in the conductive product of 80% for the class size of <0.3 mm and 56% for >1.18 mm. The concentration by gravity separation was between 64% and 75%, for 0.6–1.18 and 0.3–0.6 mm, respectively. 

The relationship between particle size and metal concentration after separation processes for lead and tantalum showed that the greater the particle size, the lower the concentration amount. Gold was found in similar quantities in the two smaller particle sizes from the different separation processes, as well for nickel. Regarding the influence of particle size on tin concentration, it was higher in intermediate particle sizes. However, aluminum had the greater concentration grade in the coarser particle size resulting from electrostatic separation. 

Considering the analyzed metallic element of Table 4, and their weight for the 75% on the enriched products mass, they were constituted by 65% of copper, 7.5% of tin, 2% of aluminum, 0.4% of nickel, 0.3% of tantalum, 0.08% of gold, and 0.01% of lead. The remaining 25% may contain fiber glass, epoxy resin, plastic, ceramic, and other metals not analyzed. In terms of price, gold represents 82% of the enriched product total price, followed by Cu (11%), Sn (5%), and Ta (1.5%). The presence in high concentrations of metals and their market value are the impetus for the development of an enrichment and recovery system of metals in a PCB. 

The metallic fraction, also considering the magnetic product, represents 35.3% of the products yield, and according to Table 1: the composition of a PCB, it probably means a high-grade PCB type, having higher concentrations of metals. 

## 4. Conclusions

Printed circuit boards are present in almost all WEEE, being a material with high metals concentrations. They represent a significant fraction of the economic value of the total electronic waste, making PCB scrap economically attractive for recycling. It conserves natural resources as it prevents new minerals from being extracted, and it is a great contribution to the circular economy.

The studied process was composed of pre-processing, formed by dismantling, comminution, and classification of the material in different granulometric sizes. Then, magnetic, gravity, and electrostatic separation were performed and chemically evaluated.

The found weight loss during all the processes was about 15% of the initial weight. During physical separation processes, losses can reach amounts of 10–35% [34]. To increase the efficiency of the mechanical pre-treatment plants, a material loss evaluation during the shredding and separation steps would be necessary to analyze in further works.

From gravity separation, the heavy fraction showed a metallic concentration of 76% for the class size of 0.6–1.18 mm and 89% for the 0.3–0.6 mm class size.

The conductive material, from electrostatic separation, had metal concentrations of about 88% for the class size <0.3 mm and 62% for the >1.18 mm one. This lower content for the coarsest grain size may be explained by the nonmetallic particles being attached to the metallic elements, in other words, the grains were not free. 

This work presents interesting results in terms of the application in industrial materials recovery processes, specifically for metals present in PCBs. From a research point of view, the integration between this first pre-treatment validation and refining chemical processes will be explored in the future with a combined approach.

## Figures and Tables

**Table 1:** Representative composition of a PCB (by wt.%) considering several types of PCBs from [15,16,17,18,19,20,21,22,23,24,25,26,27,28,29,30] and market price (USD/kg).

Metal	Weight Average (%)	Stdev (%)	Price (US$/kg)
Al	3.02	1.70	2.68
Cu	21.44	9.14	9.65
Fe	3.28	2.79	0.0994
Sn	3.14	1.65	36.79
Au	0.04	0.04	57,598.06
Pd	0.05	0.11	65,040.96
Ag	0.13	0.15	767.28
Ni	1.03	1.29	19.824
Pb	1.86	0.94	2.40
Zn	0.73	0.76	3.34
Ta	0.01	0.01	220 *
Total	34.7%		

Note: Metal price according to the London Metal Exchange (LME) (November 2021) [31]; * Tantalum price (USD 100/pound), (August 2021) [32].

**Table 2:** Weight percentage of the products obtained by magnetic separation and their yield.

Class Size	Products	% Class	Yield
>1.18 mm	Magnetic fraction	5%	0.9%
	Nonmagnetic fraction	95%	16%
0.6–1.18 mm	Magnetic fraction	2%	1.0%
	Nonmagnetic fraction	98%	49%
0.3–0.6 mm	Magnetic fraction	2.4%	0.3%
	Nonmagnetic fraction	97.6%	14%
<0.3 mm	Magnetic fraction	0.3%	0.1%
	Nonmagnetic fraction	99.7%	19%

**Table 3:** Weight percentage of the products and losses obtained by gravity separation and their yield.

Class Size	Products	%Class	Yield
0.6–1.18 mm	Heavy fraction	59%	29%
	Light fraction	41%	20%
0.3–0.6 mm	Heavy fraction	34%	5%
	Light fraction	66%	9%

**Table 4:** Weight percentage of the products and losses obtained by electrostatic separation and their yield.

Class Size	Products	Class	Yield
>1.18 mm	Conductive	64%	10%
	Nonconductive	36%	6%
<0.3 mm	Conductive	16%	3%
	Nonconductive	84%	16%

**Table 5:** Metal content (%) of the enriched products. (n.i. = not identified).

Class Size (mm)	Operation (Product)	Yield *	Concentration (%)							
			Cu	Pb	Ta	Au	Sn	Al	Ni	Others
>1.18	Electrostatic (Conductive)	10%	56.1 ± 4.3	0.001 ± 0.001	n.i.	0.01 ± 0.005	3.1 ± 2.6	3.1 ± 0.6	0.3 ± 0.1	37.3 ± 7.6
0.6–1.18	Gravity (Heavy Fraction)	29%	64.5 ± 0.4	n.i.	0.15 ± 0.15	0.05 ± 0.04	8.8 ± 0.7	1.6 ± 0.1	0.42 ± 0.02	24.4 ± 1.3
0.3–0.6	Gravity (Heavy Fraction)	5%	75.3 ± 1.25	0.03 ± 0.02	0.6 ± 0.22	0.17 ± 0.06	11.10 ± 0.92	1.58 ± 0.05	0.68 ± 0.2	10.5± 2.7
<0.3	Electrostatic (Conductive)	3%	80.5 ± 0.42	0.06 ± 0.005	0.6 ± 0.07	0.17 ± 0.02	4.55 ± 0.7	2.12 ± 0.08	0.65 ± 0.05	11.4 ± 1.32

* Yield on the total weight of products.

## Data Availability

Not applicable.
